# Romantic relationship satisfaction and phubbing: The role of loneliness and empathy

**DOI:** 10.3389/fpsyg.2022.967339

**Published:** 2022-10-21

**Authors:** Siqun Zhan, Silu Shrestha, Nian Zhong

**Affiliations:** Department of Philosophy, Wuhan University, Wuhan, China

**Keywords:** phubbing behavior, romantic relationship, relationship satisfaction, loneliness, empathy

## Abstract

This study investigates the effects of loneliness and empathy on romantic relationship satisfaction and phubbing. Loneliness plays a mediating role in romantic relationship satisfaction and phubbing. The level of empathy moderates these mediating effects. Five hundred and four Chinese adults completed tests of romantic relationship satisfaction, phubbing, loneliness, and empathy. The results show that romantic relationship satisfaction is negatively correlated with phubbing. Loneliness mediates this process. Specifically, lower romantic relationship satisfaction leads to more phubbing by increasing loneliness. Our study also shows that the mediating relationship is moderated by the level of empathy. To be more specific, the higher the level of empathy, the stronger the impact of romantic relationship satisfaction on loneliness, and the more phubbing individuals exhibit.

## Introduction

As a social animal, there is a basic need for human beings to participate in social interaction and construct their relationship network. For most adults, romantic relationships are an essential part of the daily social network. A satisfying romantic relationship can increase trust and happiness between partners (Robles et al., 2014). There is also a significant increase in individual distress when a relationship brakes up (Tashiro and Frazier, 2003). Studies have found that when relationship satisfaction is low, individuals show lower relationship commitment, care more about the cost of relationship investment (Rusbult, 1980), and seek more alternative relationships. “Therefore, it stands to reason that relationship satisfaction is associated with many negative behaviors.

Most of the attention on negative behaviors is focused on those with high intensity and great harm, such as domestic violence, and dating violence (Kaura and Lohman, 2007; Ramezani et al., 2015), while there are few studies on common but easily ignored negative behaviors, such as phubbing. But it is important to pay attention to these behaviors because the damage they cause is often long-term.

This study focuses on the relationship between romantic relationship satisfaction and phubbing behavior. The mechanism and boundary conditions are also discussed. In this article, loneliness and empathy have been studied as the mechanisms that explain how romantic relationship satisfaction affects phubbing behavior.

## Literature review

### Romantic relationship satisfaction and phubbing behavior

In this section, we first briefly review the concepts, causes, and previous related studies of romantic relationship satisfaction and phubbing behavior, then focus on the theoretical derivation of the possible correlation between romantic relationship satisfaction and phubbing behavior. Accordingly, a hypothesis is proposed.

Romantic relationship satisfaction is a key index used to measure the quality of a romantic relationship. Romantic relationship satisfaction can be defined as a person’s internal evaluation of their partner’s positive feelings and the attractiveness of their relationship (Rusbult, 1983). It represents an individual’s perception and evaluation of the current state of romantic relationships (Collins and Read, 1990).

A summary of previous studies finds that romantic relationship satisfaction is related to individual emotion and behavior. Relationship commitment, reward, and investment cost are significantly correlated with satisfaction (Sabatelli, 1988). The level of relationship stability can positively predict satisfaction (White, 1999). Dissatisfaction will also lead to a higher level of perceived relationship substitution (Attridge et al., 1995). Romantic relationship satisfaction is also a predictor of partner violence. In recent years, researchers have focused on the type of personal traits and relationship processes that lead to happier, more satisfying, and fulfilling relationships and begin to explore the relationship between satisfaction and negative behavior.

Phubbing is a phenomenon that has arisen with the popularity of smartphones. The word is derived from two words: phone and snubbing. Specifically, phubbing referred to neglecting someone else by glancing at it or using a smartphone from time to time during a face-to-face conversation (Karadağ et al., 2015). Karadağ et al. (2015) indicate that phubbing is associated with mobile addiction and with deprivation in situations of being far from one’s phone; they also indicate that the mobile phone is used as a tool helping in situations of loneliness, anxiety, and worry. Phubbing is not only a habit but also an avoidance behavior.

In some cases, people may deliberately use mobile phones to refuse to communicate with those around them (Chotpitayasunondh and Douglas, 2016). Two studies of American adults from various walks of life show that boss phubbing has a negative impact on employees’ job performance, mainly through supervisory trust and job satisfaction (Roberts and David, 2020). Yasin et al. (2020) suggest that employees who believe their supervisors use the phone more frequently in their interpersonal interactions report higher feelings of social exclusion in these interactions, which predicts lower organization-based self-esteem. In the family realm, research has found that parental phubbing behavior is highly correlated with children’s phone addiction (Xie et al., 2019). Parental phubbing behavior also leads children to perceive less parental warmth and more parental rejection, thus increasing the risk of depression (Xie and Xie, 2020). Roberts and David (2016) find that phubbing could negatively predict life satisfaction and depression levels, and a partner’s phubbing behavior has a negative impact on romantic relationship satisfaction. This behavior is more common in couples, especially if one partner is not satisfied with the relationship.

Research on phubbing has focused on its effects too. Research has shown that phubbing produces negative, resentful emotional responses (Guazzini et al., 2021) that lead people to perceive their interactions as of lower quality (Rainie and Zickuhr, 2015) and make them less trusting of their interaction partners (Cameron and Webster, 2011). The factors such as dissatisfaction about interaction with partner (Abeele et al., 2016) and jealousy about interaction partner’s mobile phone use (Krasnova et al., 2016) cause a significant increase in discouragement (Roberts and David, 2016). These factors lead to a decrease in friendship quality and a feeling of low levels of intimacy with the interactive partner (Misra et al., 2016).

Some studies have also looked at the causes of phubbing. Studies have found that internet addiction, lack of self-control, and other factors can significantly predict phubbing (Chotpitayasunondh and Douglas, 2016; Davey et al., 2018; Guazzini et al., 2019). Rahman et al. (2022) argue that phubbing is indicated by a range of factors that cannot be attributed to addiction (e.g., age, social anxiety, possession of ICT services). Phubbing has also been found to be significantly associated with negative emotional states, such as boredom, fear of missing out, anxiety, and depression (Dayapoglu et al., 2016; Elhai et al., 2016, 2018; Bolkan and Griffin, 2017). In addition, the prevalence of multitasking also leads to more phubbing behavior (Vorderer et al., 2017). Phubbing has attracted much attention from researchers as a new phenomenon, but its mechanism and conditions are not completely clear. This study examines how romantic relationship satisfaction affects phubbing behavior.

According to the need to belong theory, humans have a universal drive to form and maintain lasting, positive, and vital relationships. This desire needs to meet two criteria: first, frequent and enjoyable interactions with a small number of people; second, these interactions must demonstrate an emotional concern for each other’s well-being (Baumeister and Leary, 2017). Interactions with a constantly changing sequence of partners will be less satisfactory than repeated interactions with the same person, and relatedness without frequent contact will also be unsatisfactory. A lack of belonging constitutes severe deprivation and causes various ill effects. Moreover, this basic interpersonal motivation drives many human actions, feelings, and thoughts. When this motivation is not addressed, people feel a lack of belonging, leading to severe deprivation and ill effects. A romantic relationship is one of the crucial sources of belonging for adults. When the satisfaction of a romantic relationship is low, people will feel a decreased sense of belonging, and thus they will seek a new sense of meaning and belonging. With the development of communication technology, smartphones have become an indispensable part of people’s life. Smartphones promote social interaction, making it easy for people to communicate with others regardless of time and place (Turkle, 2011). In addition, people establish and maintain their social relationships through smartphones (Gibbs et al., 2006). As a result, when people are not satisfied with their romantic relationships, they are more likely to seek a new sense of belonging, leading to more phubbing behaviors.

Social relationships are powerfully associated with human health and well-being. Socially isolated ones suffer many difficulties, from emotional pain to increased risk for illness and death (House et al., 1988). By contrast, those with a rich social network and satisfying close relationships enjoy attenuated stress-related autonomic and hypothalamic–pituitary–adrenal (HPA) axis activity (Flinn and England, 1997; Lewis and Ramsay, 1999; Eisenberger et al., 2007). This group of people also have a lower risk for physical and psychological maladies (Moak and Agrawal, 2010). The social baseline theory can explain the reason. According to social baseline theory, the human brain expects relationships characterized by interdependence, common goals, and shared concerns (Beckes and Coan, 2011). For some individuals, violating this expectation increases cognitive and physical effort. The brain perceives fewer resources available and prepares the body to conserve or invest heavily in its energy. This increase in cognitive and physical effort often leads to acute and chronic pain, negatively affecting health and well-being. When people are dissatisfied with romantic relationships, individuals perceive some degree of relationship breakdown, which increases individual cognitive and physical efforts. This breakdown also requires individuals to redefine their separate selves, which means greater risk, greater effort, and greater loneliness. According to risk distribution and load sharing in the social baseline theory, individuals will feel negative emotions such as energy overconsumption, loneliness, and insufficient energy in an unsatisfactory romantic relationship, inducing individuals to seek their energy supplement (Coan and Sbarra, 2015). Such as expanding new social circles, immersing yourself in virtual worlds, or more phubbing.

Based on the literature review and theoretical derivation, we believe there is a correlation between romantic relationship satisfaction and phubbing behavior. We propose the following hypothesis:

*Hypothesis1*: Romantic relationship satisfaction will be negatively correlated with phubbing behavior.

### Loneliness as a mediator

After a discussion on the relationship between romantic relationship satisfaction and phubbing behavior, in this section, we analyze the possible influence of loneliness. Firstly, we sort out the concept of loneliness, review the related research on loneliness, romantic relationship satisfaction, and phubbing behavior, summarize the previous research, and deduce the possible effect of loneliness on romantic relationship satisfaction and phubbing behavior according to the media dependence theory. Accordingly, the hypothesis is put forward.

Loneliness is a universal human experience. Weiss (1973) proposes that loneliness includes emotional loneliness and social loneliness. Emotional loneliness usually occurs when an individual lacks a close spouse or partner or is dissatisfied with the relationship. Social loneliness occurs when an individual does not have appropriate social relationships or perceives limited social support. Chalise et al. (2007) argue that loneliness is complex psychology about emotions and experiences. Muyan et al. (2016) point out that loneliness is a negative emotional state closely related to individual mental health and behavior.

Loneliness is the negative experience of a discrepancy between the desired and actual personal network of relationships. People feel lonely when they perceive a difference between the level of intimacy they desire in social relationships and the level of intimacy they experience (Hartung and Renner, 2014). Everyone has an internal need to belong and be accepted by society, and this need to belong is a powerful and universal motivation. Lack of attachments is linked to various ill effects on health, adjustment, and well-being. The belongingness hypothesis is that human beings have a pervasive drive to form and maintain at least a minimum quantity of lasting, positive, and significant interpersonal relationships. Satisfying this drive involves two criteria: First, there is a need for frequent, affectively pleasant interactions with a few other people. Secondly, these interactions must take place in the context of a temporally stable and enduring framework of affective concern for each other’s welfare (Baumeister and Leary, 2017).

Studies have found that there is a correlation between romantic relationship satisfaction and loneliness. A sample survey conducted by the Australian Unity Wellbeing Database shows that interpersonal satisfaction is negatively correlated with loneliness to a moderate degree. The less satisfied a person is with their relationship, the lonelier they will feel (Mellor et al., 2008). Flora and Segrin (2000) find similar results in their research on the relationship between relationship development, romantic relationship satisfaction, and loneliness. Results from 100 participants in relationships and 100 who have recently broken up support the finding that romantic relationship satisfaction is negatively associated with loneliness. Other studies have found that romantic relationship satisfaction can mediate between attachment and loneliness, and romantic relationship satisfaction is negatively correlated with loneliness (Pereira et al., 2014). A study of 305 cohabiting undergraduates shows that loneliness is negatively correlated with romantic relationship satisfaction (Lawal and Okereke, 2021). Mund and Johnson (2021) conducted an 8-year study of 2,337 couples to investigate the role of loneliness in predicting future relationship satisfaction. Their results support the finding that loneliness is negatively associated with relationship satisfaction.

Loneliness is also linked to phubbing behavior. Media Dependency Theory can explain the relationship between loneliness and phubbing. The original purpose of media dependence theory is to conceptualize the context and sociological concepts of large social systems (Ball-Rokeach and DeFleur, 1976). The theory has been used to recently explain emerging phenomena such as social media. The core hypothesis of this theory is that the body has a sense of dependence on the media and meets some needs, and achieves some goals by acquiring media information (Ball-Rokeach, 1998). Karadağ et al. (2015) find that social media addiction is significantly related to phubbing behavior. This result meant phubbers are more likely to access social media and get information from their smartphones. Given the high correlation between social media and phubbing behavior, media dependence theory can be used to explain the relationship between loneliness and phubbing behavior.

According to media dependence theory, individuals’ media dependence can be divided into three types, which can be used to identify the importance of media to individuals. The first is the need for surveillance, where people rely on the media for information about their social environment. Secondly, social utility is the need to act effectively and meaningfully in the social world. The last need is fantasy escape, which means that when people feel at a loss, they rely on media to escape from the social environment (Jung, 2017). In the current study, the need for surveillance can be used to explain the relationship between loneliness and phubbing behavior (Ang et al., 2019). As mentioned earlier, lonely individuals experience a sense of loss, fear of social exclusion, ignorance about current phenomena in their social environment, and a need to make new social connections. As a result, they are more likely to exhibit more phubbing, such as learning about other People’s Daily lives through social media to create a sense of being in a group. In addition, the fantasy-escape need can also explain the relationship between loneliness and phubbing behavior. The decrease in romantic relationship satisfaction leads to a higher level of loneliness, which leads to a stronger need to escape from the current environment than before, leading to more phubbing.

The study by Haigh (2015) found that lonely individuals are more likely to interact with others through smartphones or social media than face-to-face communication with others. For example, a lonely individual prefers to stay at home and relies on social media to get information about the outside world rather than having face-to-face conversations with others. Therefore, the use of smartphones provides users with the opportunity to avoid face-to-face interaction with others and provides users with the opportunity to observe others without having any conversation with others (Wainner, 2018). Therefore, phubbing behavior can meet the needs of lonely individuals to monitor and escape from the social environment simultaneously. According to Ball-Rokeach and DeFleur (1976), the more an individual relies on media to meet their needs, the more important and influential social media will be to the individual. Based on this view, we can hypothesize that individuals with high levels of loneliness use social media more and are more likely to engage in social media, leading to more phubbing behavior. Therefore, media dependence theory can provide theoretical support for this study.

Jiang et al. (2018) investigate the relationship between loneliness, individualism, and smartphone addiction among international students in China. 483 students participated in the survey. The results show that people with collectivist cultures are more likely to feel lonely than those with individualism. Due to loneliness, they are at increased risk of smartphone addiction. And this smartphone addiction can eventually lead to phubbing, as smartphone use is one of the most widely recognized tools for coping with loneliness. Bian and Leung (2014) conduct a similar study on the relationship between loneliness, shyness, and smartphone addiction among Chinese students. The study aims to determine which psychological traits are stronger predictors of smartphone addiction. The results show that loneliness can significantly predict smartphone addiction, which may be because smartphones are a medium for lonely people to engage in various social activities, such as online games, chatting, or searching for messages. It is also a typical manifestation of phubbing, where individuals are more focused on their smartphones than on face-to-face communication with their partners.

Overall, it is reasonable to assume that loneliness mediates romantic relationship satisfaction and phubbing behavior. Based on the theoretical and empirical grounds, we propose the following hypothesis:

*Hypothesis 2*: Loneliness will mediate the correlation between romantic relationship satisfaction and phubbing behavior.

### Empathy As a moderator

Based on the literature review and relevant theories, we note that empathy may play an important role in the relationship between romantic relationship satisfaction and phubbing behavior. After a brief review of the concept of empathy and related research, we discuss and hypothesize the relationship between empathy and loneliness, and form a theoretical model of the relationship between romantic relationship satisfaction, loneliness, phubbing behavior, and empathy.

Empathy is a common psychological phenomenon in interpersonal communication. There are still some differences in the definition of empathy, mainly reflected in the affective orientation, cognitive orientation, and multi-dimensional orientation of empathy. First, emotion-oriented researchers believe that empathy is an emotion-affective response. For example, Eisenberg and Strayer (1987) emphasize that empathy referred to an individual’s understanding of the emotional state of others and the expression of emotional experiences and emotional responses similar to others. Secondly, cognitive-oriented researchers believe that empathy is the cognitive-based ability to understand and judge the emotions of others. For example, Ickes (1993) believes that empathy refers to the ability of an individual to understand and evaluate the psychological feelings of others. Feshbach (1987) and Hoffman (2001) also believe that empathy is the ability to experience others’ emotions through the cognition of their internal emotional states. The above researchers all believe that empathy is mainly the identification and differentiation of others’ emotional states, and empathy is generated on this cognitive basis. Finally, multi-dimensional researchers believe that empathy includes cognitive empathy and emotional empathy. For example, Gladstein (1983) believes that cognitive empathy is the main component of empathy and refers to the ability to recognize others’ emotions and understand others’ viewpoints. In addition to understanding and recognizing other people’s emotions, we must also have empathy for other people’s feelings, that is, emotional empathy.

The two-component theory of empathy has been accepted by more and more scholars (Cui et al., 2008). However, although cognitive empathy and affective empathy are necessary components of empathy, they are different. Cognitive empathy focuses on reasoning and judging emotional states, while affective empathy is mainly about feeling and experiencing other people’s emotional states. Therefore, affective empathy can be regarded as the depth of cognitive empathy, the empathetic emotional response generated after the judgment and reasoning of emotional states.

In this study, empathy refers to the subjective experience of a naturally occurring similarity between feelings expressed by the self and others without ignoring whose feelings belong to whom. Empathy includes the emotional experience of the other person’s actual or inferred emotional state and the minimal cognition and understanding of the other person’s emotional state.

Empathy is related to interpersonal functioning, promoting prosocial behavior (Van der Graaff et al., 2018) and inhibiting aggressive and externalizing problem behaviors (Batanova and Loukas, 2012). Low empathy is instead associated with more conflicts and externalizing behavior, particularly aggression and bullying (Euler et al., 2017). Although many studies have highlighted the positive role of empathy in adaptation, studies on the relationship between empathy and loneliness have produced inconsistent results.

Higher empathy is expected to be related to higher loneliness (Decety and Lamm, 2009). Emanuela et al. also find a positive correlation between empathy and loneliness when studying the relationship between depressive symptoms and loneliness in early adolescents. Also, research by Schreiter et al. support this finding. On the contrary, studies have found that loneliness is inversely correlated with empathy (Beadle et al., 2012). Hu’s research suggests empathy served as an adaptive emotion regulation strategy developed by lonely people to effectively reduce their loneliness (Hu et al., 2020).

This study suggests that empathy can enhance the impact of romantic relationship satisfaction on loneliness, which means that individuals with high empathy will experience more intense loneliness when experiencing relationship dissatisfaction. According to Theodor Reik’s definition of the processes involved in empathy, according to Theodor Reik (1949), the model of empathy can be divided into four stages. Stage 1: Identification. Focus your attention on the other person. Stage two: internalization. Make other people’s experiences your own by internalizing them. Stage three: Reverberation. Experience the experiences of others while paying attention to your cognition and emotions. Stage 4: Detachment, from merged relationships back to independent identities. It involves both understanding of others and separation from others. In terms of the four stages, compared with individuals with low levels of empathy, individuals with high levels of empathy tend to pay more attention to their partners, making them pay more attention to their romantic relationships. Such attention to a romantic relationship will strengthen the individual’s cognitive and emotional connection to the relationship. When the individual returns from the inner relationship to the position of independent identity, he will make a stronger emotional response. Thus, individuals with high levels of empathy experience greater loneliness when romantic relationship satisfaction is low.

Based on the theoretical and empirical grounds, we propose the following hypothesis:

*Hypothesis 3*: Empathy moderates the mediating role of loneliness. The higher the level of empathy, the more significant the negative correlation between romantic relationship satisfaction and loneliness, and the more phubbing behaviors.

### The present study

**Figure 1:**
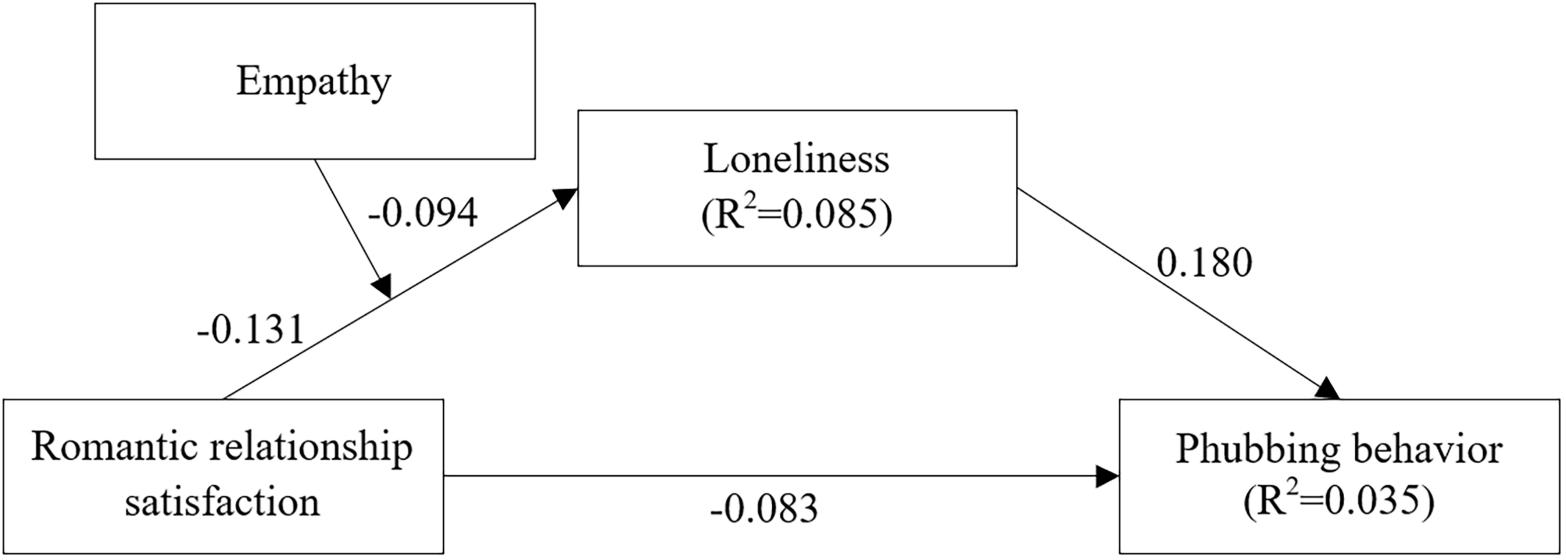
Interaction between romantic relationship satisfaction and empathy on loneliness.

In sum, the main purpose of this study is to examine whether loneliness and empathy play a role in romantic relationship satisfaction and phubbing behavior. These two questions form a conceptual model (see Figure 1).

## Materials and methods

### Participants

The sample for this study is 504 Chinese adults (319 women). In the entire sample, 40 are married and 464 adults are unmarried but in a relationship; 295 live in the same city as their partner and 209 are in a long-distance relationship. The age group ranges from 18 to over 35. 87.9% are aged between 18 and 25, 10% are aged between 26 and 34, and 1.8% are over 35. Of the total sample, 7.5% say they had been together for more than 5 years, 13% say they have been together for 3–5 years, 26% say they have been together for one to 3 years, 19% say they have been together for 6 months to 1 year, and the remaining 35% say they have been together for less than 6 months.

### Measures

#### Romantic relationship satisfaction

In this study, a brief version of the Quality of Relationship Index (QRI) revised by Patrick et al. (2007) is adopted to measure romantic relationship satisfaction. The questionnaire is a common measurement tool used by researchers at home and abroad. We use standard translations and back translations to produce the Chinese version containing six questions. A representative item is: “I feel like I am with my partner as a whole.” The questionnaire uses a 7-point Likert scale, with 1 representing “strongly disagree” and 7 representing “strongly agree.” The higher the score, the higher the satisfaction of the relationship. In this study, the Cronbach’s α is 0.94 and the structure validity is 0.90.

#### Phubbing behavior

This study measures phubbing by revising the partner phubbing behavior prepared by Roberts and David (2016). First, standard translations and back translations are used to generate the Chinese version. Then, we change the scale from third-person to first-person since we measured the participants’ own phubbing behavior. The revised scale includes nine items: “I glance at my phone when I’m talking to my partner.” Items are assessed on a 5-point Likert scale ranging from 1 (never) to 5 (always). The higher the score, the higher the frequency of phubbing when they are with their partner. The reliability and validity of the revised scale are measured. Cronbach’s α is 0.84 and structure validity is 0.87 for the current sample.

#### Loneliness

Loneliness is assessed using the Loneliness Scale developed by Russell (Russell, 1996). The scale consists of 20 items, including nine reverse scoring items, such as “I feel connected to people around me.” Participants rated each item on a 7-point Likert scale ranging from 1 (strongly disagree) to 7 (strongly agree). Finally, when all the scores are added up, a higher score indicates a higher level of loneliness. The reliability and validity of the revised scale are measured. Cronbach’s *α* is 0.90 and structure validity is 0.92 for the current sample.

#### Empathy

In this study, empathy is measured by a locally revised interpersonal response Scale (Davis, 1980; Zhang et al., 2010). There are 22 items in the Chinese version of the scale, and a representative item is: I am a rather soft-hearted person. Items are evaluated on a 5-point Likert scale, ranging from 1 (inappropriate) to 5 (very appropriate). A higher score indicates a higher level of empathy. In the current study, Cronbach’s α is 0.80 and the structure validity is 0.89.

### Procedure

This study has received ethical approval from the Ethics Committee of the corresponding author’s university. Participants fill out online questionnaires about phubbing behavior, romantic relationship satisfaction, empathy, and loneliness. The hyperlinks of the questionnaire are posted *via* WeChat, QQ, and email. Only those who fill out the consent form had access to the questionnaire. This approach has been used successfully by other studies that collect data online (Meyerson and Tryon, 2003).

### Statistics

SPSS23.0 is used to calculate descriptive statistics and correlation analysis. All items of questionnaires are summed to get the total points. Considering that some variables are not normal distributions, we reported the Spearman correlation coefficients. Bonferroni correction is conducted to correct for multiple testing.

Romantic relationship satisfaction, loneliness, empathy, and phubbing behaviors are all measured using a self-reported questionnaire. To examine the effect of the common method bias, we conduct the Hamann single factor test. Principal component analysis is performed on the measurement tools of the above variables.

The mediating effect of loneliness on the relationship between romantic relationship satisfaction and phubbing behavior is tested through a bias-corrected bootstrapping method (N = 5,000) which tests the significance of the mediating effect. Model 4 in the PROCESS is selected to test the mediation effect. First, the variables are standardized. Then, gender, age, love duration, and love distance are controlled, and the mediating effect of loneliness on romantic relationship satisfaction and phubbing behavior is tested.

In this study, the bias-corrected bootstrapping method (N = 5,000) is used to test the moderating effect of empathy on romantic relationship satisfaction and loneliness. According to the theoretical model of this study, Model7 in SPSS macro compiled by Hayes (2012) is used to test the moderated mediation model.

## Results

### Common method deviation

The result shows that the variation explained by the first factor is only 13.64%, less than 40%, indicating that the present study is probably not pervasively affected by standard method deviations (Podsakoff et al., 2003).

### Bivariate analyses

Correlations between study variables are presented in Table 1. The results show a significant negative correlation between romantic relationship satisfaction and phubbing behavior. It turns out exactly as we predicted. Thus, Hypothesis 1 is supported. There is also a significant negative correlation between romantic relationship satisfaction and loneliness. Namely, the lower the romantic relationship satisfaction, the higher the loneliness level. Loneliness is positively correlated with phubbing behavior. In other words, the lonelier the person felt, the more phubbing behaviors occur. Empathy is not significantly correlated with romantic relationship satisfaction, loneliness, and phubbing behavior.

**Table 1:** Descriptive statistics and correlations among variables of interest.

	*M*	SD	1	2	3	4
Romantic relationship satisfaction	5.86	1.16	1	−0.26^**^	0.09	−0.15^**^
Loneliness	2.12	0.53		1	−0.02	0.15^**^
Empathy	3.17	0.50			1	0.00
Phubbing behavior	2.75	0.82				1

* *p* < 0.05;

** *p* < 0.001.

### Test of the mediation model of loneliness

The results show that after adding control variables as covariates, romantic relationship satisfaction predicts phubbing behavior significantly (*t* = −3.289, *p* < 0.05), and the direct predictive effect of romantic relationship satisfaction on phubbing behavior is still significant when the mediators are included (*t* = −2.569, *p* < 0.05; see Table 2). The romantic relationship satisfaction negatively predicts loneliness (*t* = −5.488, *p* < 0.01), and loneliness also positively predicts phubbing behavior (*t* = 2.445, *p* < 0.01). In addition, the upper and lower limits of bootstrap contain a 95% confidence interval. The influence of direct effect romantic relationship satisfaction on phubbing behavior and mediating effect of loneliness does not contain 0, indicating that romantic relationship satisfaction can negatively predict phubbing and phubbing through the mediating effect of loneliness. The direct effect (−0.093) and indirect effect (−0.023) account for 80.17 and 19.83% of the total effect (−0.116), respectively. Therefore, Hypothesis 2 is supported (Table 3).

**Table 2:** Mediation model test.

Variable	Loneliness			Phubbing behavior		
	*β*	SE	*t*	*β*	SE	*t*
Constant	2.984	0.194	15.414^**^	2.488	0.378	6.579^**^
Love duration	−0.013	0.018	−0.722	0.007	0.028	0.241
Love distance	−0.006	0.052	−0.113	0.118	0.081	1.453
Age	−0.012	0.077	−0.152	−0.237	0.121	−1.968
Gender	0.037	0.052	0.703	−0.040	0.082	−0.484
Romantic relationship satisfaction	−0.123	0.022	−5.488^**^	−0.116	0.035	−3.289^*^
Loneliness				0.185	0.076	2.445^**^
*R* ^2^	0.080			0.055		
*F*	5.280^**^			3.075^**^		

* *p* < 0.05;

** *p* < 0.01.

**Table 3:** Mediating effect analysis.

	Effect	SE	BootLLCI	BootULCI	Effect proportion
Total effect	−0.116	0.035	−0.186	−0.047	
Direct effect	−0.093	0.036	−0.165	−0.022	80.17%
Indirect effect	−0.023	0.014	−0.055	−0.002	19.83%

* *p* < 0.05;

** *p* < 0.01.

### Test of the moderated mediation model of empathy

The results (See Tables 4 and 5) show that after adding control variables as covariates the product term of romantic satisfaction and empathy predicted loneliness significantly after empathy is added into the model (loneliness: *B* = –0.089, *t* = −2.606, *p* < 0.01), indicating that empathy moderates the predictive effect of romantic relationship satisfaction on loneliness.

**Table 4:** Moderated mediation model test.

Variable	Phubbing behavior			Loneliness		
	*β*	SE	*t*	*β*	SE	*t*
Constant	2.589	0.210	12.344^**^	0.031	0.134	0.231
Love duration	0.018	0.027	0.656	−0.014	0.017	−0.819
Love distance	0.138	0.081	1.700	−0.014	0.051	−0.273
Age	−0.058	0.087	−0.673	0.031	0.056	−0.549
Gender	−0.041	0.083	−0.490	0.056	0.053	1.055
Romantic relationship satisfaction	−0.086	0.036	−2.373^**^	−0.133	0.023	−5.853^**^
Loneliness	0.152	0.077	1.970^**^			
Empathy				0.012	0.050	−0.211
Romantic relationship satisfaction × empathy				−0.089	0.034	−2.606^**^
*R* ^2^	0.051			0.091		
*F*	2.870^**^			6.091^**^		

* *p* < 0.05;

** *p* < 0.01.

**Table 5:** The moderating effect of empathy on the first half.

Empathy	Effect	SE	*t*	BootLLCI	BootULCI
−0.502	−0.088	0.026	−3.401**	−0.139	−0.037
0.000	−0.133	0.023	−5.853**	−0.177	−0.088
0.502	−0.177	0.031	−5.754**	−0.238	−0.117

* *p* < 0.05;

** *p* < 0.01.

**Figure 2:**
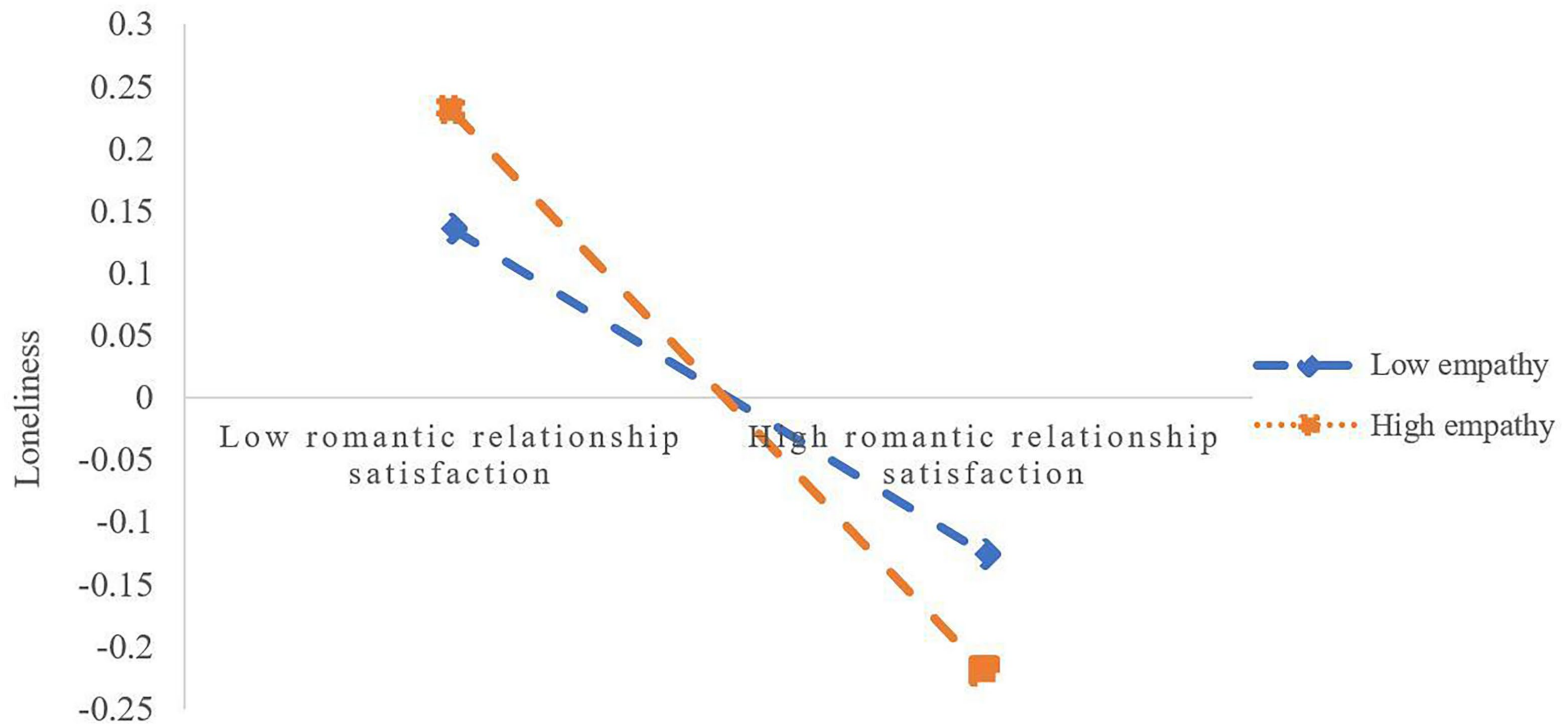
The proposed moderated mediation model.

Furthermore, simple slope analysis (see Figure 2) shows that romantic relationship satisfaction has a significant negative predictive effect on loneliness (Effect = −0.088, *t* = −3.401, *p* < 0.01) for participants with low empathy (M-1SD); However, for participants with high level of empathy (*M* + 1SD), the negative predictive effect of romantic relationship satisfaction on loneliness is significantly enhanced (Effect = −0.177, *t* = −5.754, *p* < 0.01), indicating that with the increase of empathy level, the predictive effect of intimate relationship satisfaction on loneliness gradually increased. In addition, at the three levels of empathy, the mediating effect of loneliness also increases (see Table 5).

## Discussion

In recent years, many studies have focused on romantic relationship satisfaction and phubbing behavior (Krasnova et al., 2016; Roberts and David, 2016; Cizmeci, 2017; Halpern and Katz, 2017; Wang et al., 2021). However, previous studies have not reached a consensus on the relationship or internal mechanism between the two. Meanwhile, according to the current research progress, we can find that there is indeed a pretty complex relationship between romantic relationship satisfaction and phubbing. The results of this study enrich the understanding of this issue in the following aspects. First, the relationship between romantic relationship satisfaction and phubbing behavior is mediated by loneliness. Reduced romantic relationship satisfaction leads to increased loneliness, so individuals exhibit more phubbing behaviors when dealing with their partners. Second, the above mediation model is further moderated by the level of empathy. For individuals with high levels of empathy, decreased romantic relationship satisfaction will lead to stronger loneliness and more phubbing behavior than those with low levels of empathy, which is also consistent with our hypothesis.

In addition, this study finds that the decline of romantic relationships aggravates phubbing behavior by increasing the loneliness of individuals, supporting hypothesis 2. Specifically, individuals with intense feelings of loneliness may show more avoidance of face-to-face chatting and are unwilling to engage in too much self-disclosure and meaning-seeking in reality. Therefore, they may be more likely to use phubbing behavior to monitor information from the outside world and escape from the real social environment and seek more social connections on social networks to create a sense of being in a group. No other studies have directly reached this conclusion before, so this makes us the first to propose a comprehensive model that helps to understand the causes and mechanisms of phubbing behavior and extends existing theories on phubbing behavior.

We find that participants with high levels of empathy reported feeling lonelier in unsatisfactory romantic relationships than those with low levels of empathy. Hypothesis 3 is supported. This finding provides a new perspective to explain the relationship between romantic relationship satisfaction and loneliness. Previous research on empathy and loneliness has found inconsistent results. Hu et al. (2020) research found that empathy is negatively correlated with loneliness. The opposite result is found in Decety and Lamm (2009) study, which shows that empathy level is significantly positively correlated with individual loneliness. In this study, no significant correlation is found between empathy and loneliness. However, the focus of this study is on whether empathy plays a moderating role. Therefore, this study extends the previous research to examine the moderating effect of empathy level on romantic relationship satisfaction and phubbing behavior. Consistent with our hypothesis, empathy levels modulate this association. This finding further extends the results of previous studies. It fills in the gaps of previous studies, providing for the first time the moderating effect of empathy level on romantic relationship satisfaction and phubbing behavior, thus expanding the research boundary of the romantic relationship field.

High levels of empathy will strengthen the negative correlation between romantic relationship satisfaction and loneliness because individuals with high empathy tend to be more invested in a romantic relationship, show more attention and dependence on their partner, and have more involvement. Thus, individuals with high levels of empathy experience greater feelings of insecurity and separation and exhibit more loneliness when romantic relationship satisfaction declines. Even though some of their attention is still focused on romantic relationships, individuals are more likely to use smartphones to seek a new sense of meaning and escape from real social situations due to increased loneliness. Thus, they exhibit more phubbing behaviors. In contrast, low levels of empathy reduced the negative impact of romantic relationship satisfaction on loneliness. Individuals with low empathy may not be able to empathize with their partner fully. Or, they may not be able to focus on their own emotional experience and their partner’s emotional experience, so they may not internalize the emotional experience of others. Thus, for participants with low levels of empathy, a decrease in romantic relationship satisfaction does not result in a particularly intense experience of loneliness, nor does it result in more phubbing behavior.

## Limitations and future directions

Future researchers should note the following limitations of this study. First of all, the measurement method used in this study is retrospective self-report. Although the standard method bias test is carried out in this study, the social approval effect may inevitably be affected. Second, the cross-sectional study design is adopted in this study, and the results can only show the significant correlation between various variables, and no causal inference can be drawn. Therefore, the method reported by others or cross-lag research can be considered in future studies. Third, some of the effects observed in this study are not large enough. Many factors may influence an individual’s phubbing, and romantic relationship satisfaction may be just one of them, rather than the determining factor. But that is not to say the relationship between romantic relationship satisfaction and phubbing is not worth paying attention. Fourth, this study only examines the impact of individual romantic relationship satisfaction on their bowing behavior. However, when a romantic relationship satisfaction is low, two people at the heart of a relationship often feel the satisfaction, so if both partners in this kind of circumstance respond similarly, they will react differently. The reason and mechanism of these needs further research. Finally, this study discusses the mechanism between romantic relationship satisfaction and bowing behavior but further exploration is needed to determine whether other reasons besides loneliness explain the occurrence of bowing behavior and whether bowing behavior is an avoidance behavior or a kind of revenge behavior.

## Contributions

Despite the limitations mentioned above, this study is still an important contribution to the field of romantic relationships. From a practical point of view, our study is helpful to timely detect the problem of decreased satisfaction between partners from the explicit manifestation of bowed head behavior and timely design effective measures for psychological intervention to improve romantic relationship satisfaction. Therefore, the finding that intimate relationship dissatisfaction positively affects phubbing behavior is of great significance. In addition, from a theoretical perspective, this study extends previous studies to examine the mediating role of loneliness in the relationship between romantic relationship satisfaction and bowed behavior and the moderating role of empathy in the relationship. The results of this study help to explain the potential mechanisms and pathways between romantic relationship satisfaction and bowing behavior, and explain when relationship dissatisfaction affects bowing behavior. It extends upon research on the antecedents of phubbing by further highlighting some of the potentially negative consequences of dissatisfied romantic relationship. We anticipate this to be a fruitful line of research as smartphones become more and more connected to people’s lives.

## Conclusion

In summary, the current study investigates the relationship between romantic relationship satisfaction and phubbing behavior. It extends previous literature by examining the moderating and mediating effects of empathy and loneliness in this relation.

Our study confirms that this relationship is mediated by feelings of loneliness. In other words, the negative correlation between romantic relationship satisfaction and phubbing behavior is realized by increasing individual loneliness. In addition, the mediation model is also moderated by empathy. Specifically, for adults with high levels of empathy, the lower their romantic relationship satisfaction, the lonelier they are. In contrast, for adults with low empathy, the connection becomes less important.

## Data availability statement

The original contributions presented in the study are included in the article/Supplementary material, further inquiries can be directed to the corresponding authors.

## Ethics statement

The studies involving human participants were reviewed and approved by Wuhan University. The patients/participants provided their written informed consent to participate in this study.

## Author contributions

SZ provided research ideas for this manuscript, completed data collection and analysis, and wrote the manuscript. NZ proposed the revision of the manuscript. SS proofread the grammar and words of the manuscript. All authors contributed to the article and approved the submitted version.

## Conflict of interest

The authors declare that the research was conducted in the absence of any commercial or financial relationships that could be construed as a potential conflict of interest.

## Publisher’s note

All claims expressed in this article are solely those of the authors and do not necessarily represent those of their affiliated organizations, or those of the publisher, the editors and the reviewers. Any product that may be evaluated in this article, or claim that may be made by its manufacturer, is not guaranteed or endorsed by the publisher.
